# The prevalence, risk factors analysis and evaluation of two diagnostic techniques for the detection of *Cryptosporidium* infection in diarrheic sheep from Pakistan

**DOI:** 10.1371/journal.pone.0269859

**Published:** 2022-07-08

**Authors:** Naimat Ullah Khan, Tahir Usman, Mian Saeed Sarwar, Hazrat Ali, Ali Gohar, Muhammad Asif, Fazli Rabbani, Rifat Ullah Khan, Nighat Sultana, Nazir Ahmad Khan, Muhammad Mobashar, Assar Ali Shah, Metha Wanapat

**Affiliations:** 1 Department of Clinical Medicine and Surgery, University of Veterinary and Animal Sciences, Lahore, Pakistan; 2 College of Veterinary Sciences and Animal Husbandry, Abdul Wali Khan University, Mardan, Pakistan; 3 Institute of Continuing Education and Extension, University of Veterinary and Animal Sciences, Lahore, Pakistan; 4 College of Veterinary Science, Faculty of Animal Husbandry and Veterinary Sciences, The University of Agriculture, Peshawar, Pakistan; 5 Department of Biochemistry, Hazara University Mansehra, Mansehra, Khyber Pakhtunkhwa, Pakistan; 6 Department of Animal Nutrition, The University of Agriculture, Peshawar, Peshawar, Khyber Pakhtunkhwa, Pakistan; 7 Department of Animal Science, Faculty of Agriculture, Tropical Feed Resources Research and Development Center (TROFREC), Khon Kaen University, Khon Kaen, Thailand; University of Lincoln - Brayford Campus: University of Lincoln, UNITED KINGDOM

## Abstract

**Background:**

*Cryptosporidium* spp. is recognized as an opportunistic zoonotic parasite that infects humans as well as wild and domestic animals. This enteric protozoan is a major cause of diarrhea in humans and animals and often result in death due to severe dehydration. The present study was designed to investigate the prevalence, identification of various risk factors and evaluation of sensitivity of the two diagnostic techniques for rapid and correct detection of *Cryptosporidium* infection in diarrheic sheep in Pakistan.

**Methods:**

A total of 360 fecal samples were collected and processed for detection of *Cryptosporidium* infection after proper preservation. These samples were properly stained with modified Ziehl-Neelsen acid staining and then examined under simple microscope at 100x magnification for confirmation of *Cryptosporidium* oocysts. The same samples were again processed through simple PCR for confirmation of the *Cryptosporidium spp*.

**Results:**

The age wise prevalence was detected through simple microscopy and PCR. We found highest prevalence at the age of ≤1 year followed by 1–2 years of age while the lowest prevalence was recorded at the age of ≥ 2–3 years of sheep and found significant difference between different ages (P<0.05). The sex wise prevalence showed the highest prevalence in male (♂) animals detected compared to female (♀**).** The overall prevalence was detected 27.08% and 18.80% through PCR and simple microscopy, respectively, and significant difference between two diagnostic techniques were observed (P<0.05). Considering the seasonality, the highest prevalence was recorded through simple microscopy in autumn, summer, and spring, while the lowest in winter. These results were confirmed through PCR.

**Conclusion:**

It was concluded that molecular detection is the most efficient, specific and sensitive technique for detection of *Cryptosporidium* infection than simple microscopy. Moreover sheep is the major potential source of infection to other wild and domestic animals including humans.

## 1. Introduction

*Cryptosporidium* infection is an enteric protozoan parasitic disease caused by *Cryptosporidiu*m species belonging to Phylum Apicomplexa. *Cryptosporidium* infection is caused by an obligate and intracellular protozoan causing intestinal infection in wild and domestic animals. The cases of *Cryptosporidium* infection has been reported in more than twenty two domestic animals, as well as wild species such as mammals, fish, birds and reptiles [1, 2]. *Cryptosporidium* infection carries public health significance because it has been reported in humans and large number of vertebrates such as sheep, goats, cows, dogs, cats, reptiles, fish and poultry. Moreover, this protozoan has public health significance because zoonotic transmission can occur when come in contact with any infected animals [3, 4]. *Cryptosporidium* infection (cryptosporidiosis) has been mainly recorded in wet, humid and hot weather of the year [5, 6]. *Cryptosporidium* infection is a serious threat to the immune compromised individuals and sometimes it can become chronic and even fatal infection [1, 7].

*Cryptosporidium* infection has been reported in various agro-ecological areas where it is the serious health threat to small and large ruminants. *Cryptosporidium* infection has been recorded as a stern threat to the economy world widely [8–10]. The presences of *Cryptosporidium* species (*C*. *hominis* and *C*. *parvum*) in small ruminants raise the significant of sheep in the transmission of the infection [11]. However, there is further need of epidemiological studies at molecular level to find out zoonotic species in small ruminants and their attendant for the betterment of public health significance. Generally, diagnosis is based on simple microscopic identification of oocysts; that is a big challenge because some acid fast bacteria such as *Mycobacterium* species also stain at the same time. Therefore only trained laboratory technician can only differentiate between *Cryptosporidium* and *Mycobacterium* species [12, 13].

Many researchers have also used simple technique where samples of fecal substance (1–20 gram) were mixed with concentrated solution of Sodium Chloride and then applied centrifugation. This technique is the best approach as indicated by the researchers to identify positive specimens after performing microscopy [1, 11, 13].

Therefore, it is suggested that molecular diagnostic techniques (PCR, RT-PCR) are the most fast, specific, and sensitive techniques for the detection of protozoan’s infection in various domestic and wild animals. Moreover diagnostic techniques have more advantages over simple microscopy. The molecular techniques are more sensitive and reliable for correct detection of protozoan infection [11, 14]. Therefore, the present study was designed to investigate the prevalence, risk factors analysis and to compare the sensitivity of the two diagnostic techniques (PCR vs Microscopy) for rapid and correct diagnosis of *Cryptosporidium* infection in sheep.

## 2. Materials and methods

### 2.1 Ethics statement

The study was approved by the ethics committees (No. 14101/2017) of the clinical medicine & surgery (CMS) department, and parasitology department, the University of Veterinary and Animal Sciences Lahore, Pakistan.

### 2.2 Collection and preparation of fecal samples

Three districts were selected for collection of fecal samples (Bannu, Lakki Marwat and Kohat) of southern Khyber Pakhtunkhwa, Pakistan. A total of 360 fecal samples were collected from diarrheic sheep using a convenient sampling technique. While approaching to animals, various risk factors such as ages, area, sex and season were considered and the basic information’s were entered on already designed questionnaire. The samples were collected from different age groups of sheep. Group 1 sheep were less than one year, group 2 sheep were of 1–2 years whereas, group 3 sheep were more than 2–3 years of age.

This study was continued for a period of one year. All the samples were collected from local breed of sheep in two manners, (1) freshly passed feces and (2) secondly from rectum. During direct collection, all the ethical parameters were followed and advance ethical permission was taken from university ethical committee. Secondly, prior permission was also taken from the owners of the sheep and free necessary treatment was provided to the sheep flock at the time of fecal collection. All the samples were collected from the sheep having private owners and no one was purchased. In the study area each owner has a flock of more than 300 sheep. The fecal samples of about 10 gm were collected from the rectum of the sheep or either freshly passed feces wearing gloves and then before processing the samples, all the samples were divided in two parts. One part was processed and examined through simple microscopic examination while other parts of the same samples were processed through PCR. Before the laboratory examination, all the samples were processed through flotation technique for maximum concentration of oocysts and then applied both laboratory techniques (microscopy or PCR). The flotation technique was applied to concentrate the oocysts. Formalin was added to only those samples that have to process only through conventional microscopy at the at ratio of 1:3 and stored at 4C°for 1–2 weeks till analysis while for PCR all the samples were kept without formalin. Samples were stored at -60°C for the following molecular examination [15].

### 2.3 Microscopic identification

Each fecal sample of 5gm was weighed by electric balance and then dissolved in water to form a homogenized solution. Then the solution was centrifuged at the rate of 1500 rpm for 1–2 minutes. Each slide was stained with modified Ziehl-Neelsen acid staining and confirmed the presence of *Cryptosporidium* oocysts through simple microscope at magnification of 100X [16].

### 2.4 Staining procedure (modified Ziehl-Neelsen acid fast staining)

First prepared a thin fecal smear and the slide were allowed to dry in air. After drying it was fixed in methanol for 2–3 minutes. After fixing, the smear was stained with Carbol fuchsin (15–20 minutes) and washed with the help of tape water. The acid alcohol (1%) was applied as a decolorizing agent for 2 minutes. After staining, all the slides were washed with tap water and then applied methylene blue (counter stain) for a period of 1 minute. Finally slides were rinsed with tap water and left to air dry. Each slide was examined (using oil immersion) under 100x magnification using a calibrated light microscope, as reported by Bakiret et al. [17]. All those slides where only one oocyst identified, was declared a positive case.

### 2.5 Identification and confirmation of oocysts (*Cryptosporidium*)

All the oocysts of C*ryptosporidium* were identified and diagnosed on the basis of size, shape and the keys as described by Watanabe et al. [18].

### 2.6 DNA extraction

DNA was extracted from oocysts using a method as described by Da Silva et al. [19] with minimum variations. The DNA extraction kit (GFC vivantis, USA) was utilized for disruption of the tissue of the crypto oocysts and the DNA was extracted. The quality and quantity of the extracted DNA was assessed on 2% agarose gel electrophoresis and photograph was taken by gel documentation system.

### 2.7 Amplification of DNA

The 18s rRNA was the targeted gene for the detection of *Cryptosporidium* spp. The procedure used for the amplification of a gene was according to the technique as mentioned by the Da Silva et al. [19] and Johnson et al. [20] who recognized the basic coverage sequence and primers that were used for polymerase chain reaction (PCR). The following sequence of primers was used as a Forward primers sequence: (5-AAGCTCGTAGTTGGATTTCTG- and for Reverse primer sequence (5’-TAAGGTGCTGAAGGAGTAAGG-3’). The reaction mixture was mixed with ice tar and the mixture was arranged for five reactive processes. The first step of PCR was denatureation at temperature of 94C°for 5 min followed by 35 cycles of denatureation at 94C° for30 sec, and then annealing was processed at 65C° (15–60 sec) and finally extension was performed at 72C° for 1 min. Each amplified DNA sample was properly labeled and stored at -20C° for further use. After that, the 2% gel electrophoresis was processed that was stained with ethidium bromide and was used for visualization of the samples. Finally photograph was taken in gel documentation system.

### 2.8 Statistical analysis

The collected data was subjected to the statistical analysis using the version 20 Statistical package for Social Sciences (SPSS). Prevalence rates were calculated and presented in the form of percentages (%). Data regarding with comparison of differences in prevalence for different variables and associated risk factors were analyzed using chi-square test (X^2^).

## 3. Results

The season wise, age wise, month wise and sex wise prevalence of *Cryptosporidium* infection was investigated in diarrheic sheep using simple microscopy and PCR as illustrated in various tables. The season wise data was collected and evaluated through simple microscopy and PCR which showed the highest prevalence in summer season (27.50%) followed by autumn (20.00%) and spring (18.33%), while the lowest in winter (8.33%) and similarly through PCR, the maximum prevalence was in summer (33.33%), autumn (30.00%), spring (26.66%), and winter (13.33%). We found a non-significant difference (P<0.27) (Table 1) between two diagnostic procedures.

**Table 1:** Season wise prevalence of *Cryptosporidium* infection in sheep detected by simple microscopy.

Factors	District Bannu		District Lakki Marwat		District Kohat		Overall Prevalence		
	Infected / Total Examined	Prevalence (%)	Infected/ Total Examined	Prevalence (%)	Infected/ Total Examined	Prevalence (%)	Infected/ Total Examined	Prevalence (%)*	P- value
Winter	4/40	10.00	2/40	5.00	4/40	10.00	10/120	8.33 ^c^	0.004
Spring	3/20	15.00	4/20	20.00	4/20	20.00	11/60	18.33 ^b^	
Summer	11/40	27.50	8/40	20.00	14/40	35.00	33/120	27.5^a^	
Autumn	4/20	20.00	4/20	20.00	4/20	20.00	12/60	20^ab^	
Total	22/120	72.00	18/120	65.00	26/120	85.00	66/360	18.33%	

*Mean values with different superscripts differ significantly at (*P*<0.05).

The age wise prevalence of *Cryptosporidium* infection was also determined through simples microscopy (Table 2) and PCR then both results were compared with each other (Table 5). As a result of simple microscopy, the highest prevalence was observed at the age of ≤1 year (23.13%) followed by 1–2 years (18.85%) whereas the lowest prevalence was detected at the age of ≥2–3 years (11.53%) as shown in Table 2. The comparative results of two diagnostic techniques (simple microscopic and molecular detection) used for detection of *Cryptosporidium* infection was 29.85%, 23.13% (at age of ≤1 year), 26.22%, 18.85% (1- years of age) followed by 17.30% and 11.53% (at age of ≥2–3 years), respectively and statistically found non-significant difference (P<0.264) as shown in Table 5.

**Table 2:** Age wise prevalence of *Cryptosporidium* infection in sheep detected by simple microscopy.

Age wise animals	District Bannu		District Lakki Marwat		District Kohat		Overall		
	Infected/ Total Examined	Prevalence (%)	Infected/ Total Examined	Prevalence (%)	Infected/ Total Examined	Prevalence (%)	Infected/ Total Examined	Prevalence (%)*	P-value
≤1 year age	11/44	25.05^a^	8/38	21.05^b^	12/52	23.07^a^^b^	31/134	23.13^a^	0.05
1–2 years age	9/44	20.45^a^^b^	6/42	14.28^b^	8/36	22.22^a^	23/122	18.85^a^^b^	
≥2-3years age	2/32	6.25^c^	4/20	10.23^b^	6/32	18.75^a^	12/104	11.53^b^	

^a, b, c^ Mean values with different superscripts differ significantly at (*P*<0.05).

The month wise prevalence was also detected where, the highest prevalence was found in the month of August (36.66%) followed by July (26.66%) while the lower most prevalence was observed in the month of December and January (6.66%) (Table 3). It was concluded that maximum prevalence occurs in hot months of the year than cool months. The Sex wise prevalence was also determined through simple microscopy and PCR. The microscopic results showed the highest prevalence in female sheep (18.80%) than male sheep (17.02) (Table 4). The sex wise prevalence was also determined through microscopic examination and PCR where maximum prevalence was observed in female (18.80%: 27.08%) than male (17.02%: 25.53%) (Table 5) and statistically found significant difference between two diagnostic techniques (P<0.02).

**Table 3:** Month wise prevalence of *Cryptosporidium* infection in sheep detected by simple microscopy.

Factors	District Bannu		District Lakki Marwat		District Kohat		Overall percent prevalence	
	Infected/ Total Examined	Prevalence (%)	Infected/ Total Examined	Prevalence (%)	Infected/ Total Examined	Prevalence (%)	Infected/ Total Examined	Prevalence (%)*
January	0/10	00.00	1/10	10.00	1/10	10.00	2/30	6.66^d^
February	2/10	20.00	0/10	00.00	1/10	10.00	3/30	10.66^c^
March	1/10	10.00	1/10	10.00	1/10	10.00	3/30	10.00^c^
April	2/10	20.00	3/10	30.00	3/10	30.00	8/30	26.66^b^
May	2/10	20.00	3/10	30.00	2/10	20.00	7/30	23.33^d^
June	3/10	30.00	1/10	10.00	4/10	40.00	8/30	20.00^f^
July	2/10	20.00	2/20	20.00	3/10	30.00	7/30	26.66^d^
August	4/10	40.00	2/10	20.00	5/10	50.00	11/30	36.66^a^
September	1/10	10.00	3/10	30.00	3/10	20.00	7/30	23.33^d^
October	3/10	30.00	1/10	10.00	1/10	10.00	5/30	16.66^n^
November	1/10	10.00	1/10	10.00	1/10	10.00	3/30	10.00^c^
December	1/10	10.00	0/10	00.00	1/10	10.00	2/30	6.66^d^
Total	22/120	18.33%	18/120	15.00%	26/120	21.66%	66/360	18.33%

^a, b, c^ Mean values with different superscripts differ significantly at (*P*<0.05).

**Table 4:** Sex wise prevalence of *Cryptosporidium* infection in sheep by simple microscopy.

Factors	District Bannu		District Lakki Marwat		District Kohat		Overall		
	Infected / Total Examined	Prevalence (%)	Infected /Total Examined	Prevalence (%)	Infected/ Total Examined	Prevalence (%)	Infected/ Total Examined	Prevalence (%)*	P-value
Male	6/36	18.75	4/28	14.28	6/30	20.00	16//94	17.02^a^	0.69
Female	16/84	19.04	14/92	15.21	20/90	22.22	50/266	18.80^a^	

^a, b, c^ Mean values with different superscripts differ significantly at (*P*<0.05).

**Table 5:** Comparative evaluation of the sensitivity of two diagnostic techniques (PCR & microscopy) for detection of *Cryptosporidium* infection in sheep.

Factors		No. of positive samples/total No. of samples examined by PCR	Molecular (%) prevalence	Microscopic (%) prevalence	value
Area wise prevalence	Bannu	30/120	25.00	18.33	0.19
	Lakki Marwat	22/120	18.33	15.00	
	Kohat	38/120	31.66	21.66	
Season wise prevalence	Winter	16/120	13.33	8.33	0.27
	Spring	16/60	26.66	18.33	
	Summer	40/120	33.33	27.50	
	Autumn	18/60	30.00	20.00	
Sex wise prevalence	Male	24/94	25.53	17.02	0.02
	Female	58/266	27.08	18.80	
Age wise prevalence	≤1 year of age	40/134	29.85	23.13	0.26
	1–2 years of age	32/122	26.22	18.85	
	≥2–3 years of age	18/104	17.30	11.53	

^a, b, c^ Mean values with different superscripts differ significantly at (*P*< 0.05).

## 4. Discussion

The current study showed that there is high incidence of *Cryptosporidium* infection in diarrheic sheep that were clinically characterized by shooting diarrhea and dehydration resulting in high mortality in Pakistan. Similarly other countries in Southern Asia are also at risk for the *Cryptosporidium* infection in small ruminants. The advance diagnostic technique was applied for detection of *Cryptosporidium* infection such a polymerase chain reaction (PCR). The PCR based finding of the *Cryptosporidium* infection was more sensitive method than other conventional microscopy [21].

The highest prevalence of *Cryptosporidium* infection was recorded in the summer season while the lowest prevalence was recorded in the winter season. Our results are consistent and agree with the results of other researchers and investigators who reported a strong correlation between the warm and wet seasons with the infection rate. A study [22] documented (17.3%) the highest percent prevalence in the summer season in Mazandaran province of Iran where rainfall was maximum [23]. Some other researchers also reported similar results who also reported maximum prevalence of *Cryptosporidium* infection in rainy and warm season of the year [24] that reach to the highest point in spring and summer season [25]. The highest percent prevalence was recorded in the summer season due to the reason of high intake of water and increased outdoor activities such as swimming (summer activities) during the summer season in recreational water in the form of community swimming and enhancing the chances for fecal-oral transmission [26]. Similarly adult animals produce a large volume of faeces and thus may be responsible for environmental contamination with *Cryptosporidium*, spp. as reported by Dessì et al. [27].

The previous reports of the spread of *Cryptosporidium* infection in adults’ animal have shown difference ratios ranging from 0 to 71%. Although the majority outbreak of less than 7% has been reported and this is the conclusion those adult animals make no significant contribution to the spread of *Cryptosporidium* infection [11]. In the present investigation, the simple approach of PCR was applied to determine the percentage of *Cryptosporidium* infection and found this method was more reliable and sensitive than the conventional approach.

The DNA band was discovered from 435 base pairs (BP) that confirmed the accurate amplification of the primers for the *Cryptosporidium spp*. The polymerase chain reaction (PCR) was first used in 1991 as a rapid sensitive diagnostic tool to detect *Cryptosporidium* oocysts in manure and water samples. The *Cryptosporidium* species were identified using various molecular diagnostic techniques such as plain PCR, PCR-RFLP, RT-PCR and nasal PCR methods [28]. According to the Kabayiza et al. [29], who conducted study on 112 animals having different ages 0–2.5 years and were without diarrhea. As a result only 10 animals were positive for *Cryptosporidium* infection (8.93%). This result is lower than the results obtained in Tanzania (10.4%) and Ethiopia (9.4%), respectively. The highest prevalence was also found in those countries where average rainfall was recorded like Nigeria, 38.3% [30]. It is almost low due to the fact of drinking of clear water, toilet use, less flood and better city drainage system. Also, this low prevalence may be due to use of simple microscopy as diagnostic technique than PCR [31].

The maximum molecular ratio was recorded at the age of 1 year, followed by 1–2 years of age, while the minimum ratio was recorded at the age of 3 years or above. A total of 915 fecal samples were collected from sheep farm in Italy and examined under simple microscopic examination after proper staining. As a result, maximum prevalence was found in diarrheic samples than paste or normal feces. While the genotype analysis revealed the presence of two *Cryptosporidium* species such as *C*. *parvum* and *C*. *ubiquitum*. These both consequences have health-related implications because both are *Cryptosporidium* identified species are considered to be zoonotic, and *C*. *parvum* is the 2^nd^ most wide spread cause of human diarrhea [27].

The PCR specialization provides an option to traditional analytical methods for the finding of *Cryptosporidium* oocysts in clinical and ecological models. We measure up to a simple microscopic test using a Ziehl Neelson modified acid stain washing method with a common PCR system that could even detect a single oocysts of *Cryptosporidium* and different species of protozoa based on DNA presence [6]. The molecular technique was used for detection of *Cryptosporidium* infection that showed more rapid and sensitive results than simple microscopy in diarrheic lambs, and revealed that PCR is more sensitive method for diagnosis of *Cryptosporidium* infection [32]. While using a PCR, one can easily detect *Cryptosporidium* infection even having single oocysts in a fecal sample. It’s in line with our research where diarrheal samples were obtained and found a high number of oocysts using PCR technique [33].

## 5. Conclusion

The study infers that PCR is more reliable, sensitive and can be used as a rapid diagnosis technique for detection of *Cryptosporidium* infection than conventional microscopy. Furthermore, PCR can distinguish between different types of *Cryptosporidium species* after proper Sequencing whereas simple microscopy cannot differentiate between different species. The results conclude that PCR has many advantages over simple microscopy and is a more sensitive, authentic, rapid and the most reliable technique for the detection of *Cryptosporidium* infection in sheep.

## Supporting information

S1 Fig Agarose gel electrophoresis pattern of PCR amplicons of Cryptosporidium Oocysts in (A) autumn; (B) spring; (C) summer; (D) winter. (DOC)
