# Supplementary material for: The prevalence, risk factors analysis and evaluation of two diagnostic techniques for the detection of Cryptosporidium infection in diarrheic sheep from Pakistan
Source: PLoS One. 2022 Jul 8;17(7):e0269859. doi: 10.1371/journal.pone.0269859 (PMC9269430; doi:10.1371/journal.pone.0269859)

**Supporting information**

**Figure 1: Agarose gel electrophoresis pattern of PCR amplicons of Cryptosporidium Oocysts in (A) autumn; (B) spring; (C) summer; (D) winter.**


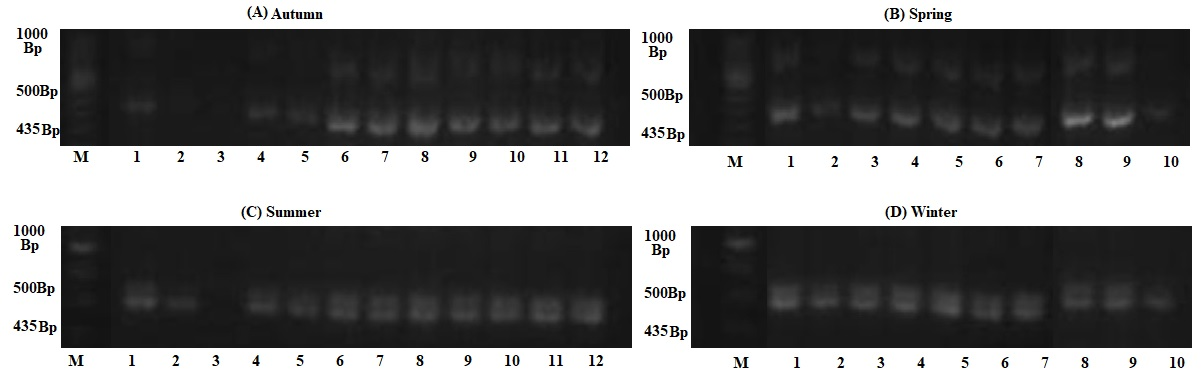

Supplement: S1 Fig — (DOC) [file pone.0269859.s001.doc]
